# PI3‐kinase/Akt pathway‐regulated membrane transportation of acid‐sensing ion channel 1a/Calcium ion influx/endoplasmic reticulum stress activation on PDGF‐induced HSC Activation

**DOI:** 10.1111/jcmm.14275

**Published:** 2019-04-02

**Authors:** Longquan Zuo, Yueqin Zhu, Lili Hu, Yanyi Liu, Yinghong Wang, Yamin Hu, Huan Wang, Xuesheng Pan, Kuayue Li, Na Du, Yan Huang

**Affiliations:** ^1^ Department of Pharmacy Hospital of Armed Police of Anhui Province Hefei China; ^2^ School of Pharmacy Anhui Medical University Hefei China; ^3^ Institute for Liver Diseases Anhui Medical University Hefei China; ^4^ The First Affiliated Hospital of USTC, Division of Life Sciences and Medicine University of Science and Technology of China Hefei China

**Keywords:** ASIC1a, ERS, HSCs, liver fibrosis, PDGF, PI3K/AKT

## Abstract

Acid‐sensing ion channel 1a (ASIC1a) allows Na^+^ and Ca^2+^ flow into cells. It is expressed during inflammation, in tumour and ischaemic tissue, in the central nervous system and non‐neuronal injury environments. Endoplasmic reticulum stress (ERS) is caused by the accumulation of misfolded proteins that interferes with intracellular calcium homoeostasis. Our recent reports showed ASIC1a and ERS are involved in liver fibrosis progression, particularly in hepatic stellate cell (HSC) activation. In this study, we investigated the roles of ASIC1a and ERS in activated HSC. We found that ASIC1a and ERS‐related proteins were up‐regulated in carbon tetrachloride (CCl_4_)‐induced fibrotic mouse liver tissues, and in patient liver tissues with hepatocellular carcinoma with severe liver fibrosis. The results show silencing ASIC1a reduced the expression of ERS‐related biomarkers GRP78, Caspase12 and IREI‐XBP1. And, ERS inhibition by 4‐PBA down‐regulated the high expression of ASIC1a induced by PDGF, suggesting an interactive relationship. In PDGF‐induced HSCs, ASIC1a was activated and migrated to the cell membrane, leading to extracellular calcium influx and ERS, which was mediated by PI3K/AKT pathway. Our work shows PDGF‐activated ASIC1a via the PI3K/AKT pathway, induced ERS and promoted liver fibrosis progression.

## 1 INTRODUCTION

Acid‐sensing ion channels (ASICs) are members of the degenerin/epithelial sodium channel family, acting as sensors for extracellular protons distributed in the nervous system and many non‐neuronal cells.^1^, ^2^, ^3^ To date, functional cloning of ASIC subunits has identified four genes (ASIC1, 2, 3 and 4) that yield at least seven ASIC isoforms: ASIC1a, ASIC1b, ASIC1b2, ASIC2a, ASIC2b, ASIC3 and ASIC4.^4^ ASIC1a is the most abundant isoform, playing important roles in a variety of physiological and pathological processes including learning, memory, fear, depression, pain, anxiety and ischaemic stroke.^5^, ^6^, ^7^, ^8^ Most ASIC1a research has focused on the peripheral nervous system and CNS neurons. Recent reports show ASIC1a is expressed in and affects non‐neuronal cells.^9^, ^10^ Although ASICs subunits possess differing ion permeability, ASIC1a is solely permeable to Ca^2+^.^11^, ^12^, ^13^, ^14^^.^

The endoplasmic reticulum (ER) is a complex and key organelle for protein folding, and lipid and carbohydrate metabolism.^15^ It is also an important Ca^2+^ storage station that regulates intracellular Ca^2+^ signalling that affects many signalling pathways.^16^, ^17^, ^18^, ^19^, ^20^ Intracellular calcium disorders affect ER functions including protein synthesis, folding and translation, which triggers endoplasmic reticulum stress (ERS) and activates the unfolded protein response (UPR).^21^, ^22^, ^23^ UPR is mediated by three ER membrane‐associated proteins, including protein kinase RNA‐like endoplasmic reticulum kinase (PERK), inositol‐requiring 1α (IRE1α) and activating transcription factor 6 (ATF6).^24^, ^25^, ^26^, ^27^ IRE1a is the most conservative branch, it regulates the activity of transcription factor X‐box binding protein (Xbp1) through recruitment of signalling molecules, splicing, and production of an active transcription factor.^28^

Liver fibrosis is a dynamic process triggered by chronic liver injury, chronic viral infections, alcoholic liver disease, autoimmune disease and hereditary metabolic disorders, which is the final common pathway in a variety of liver diseases.^29^ Hepatic stellate cells (HSCs) are the primary fibre producing cells in the liver. During inflammation or mechanical stimulation, HSCs are activated, proliferate and increase the expression of a‐smooth muscle actin (a‐SMA) and extracellular matrix (ECM).^30^, ^31^ Our preliminary experimental results showed high ASIC1a expression in liver fibrosis tissue and PDGF‐activated HSCs, and a strong correlation of increased ECM in HSCs.^32^ Aissouni et al. and Bo Duan et al. showed that PI3K/Akt signalling affects ASIC1a expression and activity via membrane transduction in neurons.^33^, ^34^ This has not been reported in liver fibrosis or HSCs. ERS is also involved in the pathogenesis of liver fibrosis, where it activates fat generation transcription factor, triggers inflammation and fibrosis and promotes fatty liver and liver fibrosis.^35^, ^36^

Given that ERS and ASIC1a both play roles in liver fibrosis pathogenesis, we analysed their effects on HSC activation and proliferation, and looked at possible signalling pathways associated with disease states.

## 2 MATERIALS AND METHODS

### 2.1 Animals

Adult male Sprague‐Dawley (SD) rats (100‐140 g) were provided by the Experimental Animal Center of Anhui Medical University (Hefei, China). All animal protocols were reviewed and approved by the University Animal Care and Use Committee of Anhui Medical University. Thirty SD rats were randomly divided into normal (n = 15) and liver fibrosis groups (n = 15). All animals were adapted 7 days prior to experiments. The liver fibrosis model was established by injecting carbon tetrachloride (CCl_4_, Shantou Xilong Chemistry Plant, China) diluted (3:2) in olive oil (4 ml of CCl_4_/kg bodyweight for the first dose, and 2 ml/kg for the remaining doses) twice weekly for 8 weeks. Control animals were treated with 2 ml/kg body weight olive oil. 35

### 2.2 Patient tissue samples

Patient tissue samples (n =30) were collected from the First Affiliated Hospital of Anhui Medical University (HeFei, China). A total of 20 liver fibrosis samples were obtained from 20 patients with liver cancer with severe liver fibrosis, and 10 normal samples were obtained from liver tissue with biliary calculi. The study was approved by the Research Ethics Committee of the Anhui Medical University, and informed consent was obtained from all patients.

### 2.3 Cell culture and transfection

Rat HSC‐T6 cells, a cell line of human liver fibroblasts (KeyGEN Bio TECH, Nanjing, China), were maintained in Dulbecco's modified Eagle's medium (DMEM, supplemented with 10% foetal bovine serum (FBS), 100 U/ml penicillin and 100 mg/ml streptomycin). HSC‐T6 cells were cultured in DMEM with 10% FBS and incubated in a humidified atmosphere 5% CO_2_ at 37°C.

Hepatic stellate cell‐T6 cells (2 × 10^5^) were grown to 60%‐80% confluence with antibiotic‐free DMEM in six‐well plates for 12 hours and then transfected with shRNA using Lipofectamine TM2000 (Invitrogen, USA) according to the instructions. Short hairpin RNA (shRNA) oligonucleotides were provided by the Shanghai Gena Pharma Corporation (Shanghai, China) for ASIC1a genes or scrambled sequences. The sequence for ASIC1a RNAi was 5’‐CACCGCCAAGAAGTTCAACAAATCGTTCAAGAGACGATTTGTTGAACTTCTTGGCTTTTTTG‐3’ (forward) and 5’‐GATCCAAAAAAGCCAAGAAGTTCAACAAATCGTCCTTGAACGATTTGTTGAACTTCTTGGC‐3’ (reverse) and the sequence for the control RNAi was 5’‐CACCGTTCTCCGAACGTGTCACGTTTCAAGAGAACGTGACACGTTCGGAGAATTTTTTG‐3’ (forward) and 5’‐GATCCAAAAAATTCTCCGAACGTGTCACGTTCTCTTAAACGTGACACGTTCGGAGAAC‐3’ (reverse).

### 2.4 Hematoxylin and eosin staining and immunohistochemistry

Tissue samples were fixed in 10% buffered formalin for 48 hours and then embedded in paraffin; 5‐μm slides were cut and dried overnight. Sections were de‐paraffinized and rehydrated prior to staining with hematoxylin and eosin and the indicated antibodies, including anti‐a‐SMA (Bioss, China, 1:100), anti‐Collagen I (Bioss, China, 1:100), anti‐ASIC1a (1:250, Alomone, USA), anti‐GRP78 (Bioss, China, 1:250,) and anti‐p‐AKT (Elabscience, China, 1:250). Samples were incubated with primary antibodies overnight at 4°C, secondary antibodies for 30 minutes. Slides were washed in PBS (Boster, Wuhan, China) 3 × 10 minutes after primary and secondary antibody incubations.

### 2.5 Immunofluorescence

Cells were fixed for 10 minutes in 4% paraformaldehyde, washed with PBS and then blocked with PBS containing 10% bovine serum albumin (BSA, Sigma, St. Louis, MO, USA) for 1 hour. Cells were incubated with primary antibody against ASIC1a (1:100, Alomone, USA) overnight at 4°C, then with a FITC‐conjugated anti‐rabbit IgG (Molecular Probes, Beijing, China) in the dark for 1 hour at 37°C. Nucleus was stained with 4,6‐diamidino‐2‐phenylindole, dilactate (DAPI; Invitrogen, Carlsbad, CA, USA) in the dark for 5 minutes, then the coverslips were imaged via an inverted fluorescence microscope (Olympus, Tokyo, Japan).

### 2.6 MTT(3‐[4,5‐dimethylthiazol‐2‐yl]‐2,5‐diphenyltetrazoliumbromide) assay

HSC‐T6 cells (5 × 10^3^) were grown to 60%‐80% confluence with DMEM in 96‐well culture plates for 24 hours. 5 mg/ml MTT agentia was added at 37°C for 4 hours before adding DMSO to dissolve formazan crystals. Cells were measured in triplicate at 490 nm wavelength using a Thermomax microplate reader (bio‐tekEL, USA). Cell viability is expressed as percentage of value in control group.

### 2.7 Quantitative real‐time reverse transcriptase polymerase chain reaction

RD SYBR^®^ qPCR Mix (Toyobo Co. Ltd, Osaka, Japan) was used according to the manufacturer's instructions. β‐actin was used as the endogenous control. The primers were designed and synthesized by Shanghai Biology Engineering Corporation according to the serial number from GenBank, shown in Table 1. Similar results were obtained from three independent samples.

**Table 1:** The primers of relative mRNAs

	Forward	Reverse
α‐SMA	5’‐CGAAGCGCAGAGCAAGAGA‐3’	5’‐CATGTCGTCCCAGTTGGTGAT‐3’
Collagen1	5’‐GATCCTGCCGATGTCGCTAT‐3’	5’‐TGTAGGCTAGCTGTTCTTGCA‐3’
GRP78	5’‐CTGTCAGCAGGACATCAAGTTC‐3’	5’‐TGTTTGCCCACCTCCATTATCA‐3’
IRE1	5’‐TGGACTGGCGGGAGAACATC‐3’	5’‐ GAGCTCCCGGTAGTGGTGTC‐3’
XBP1	5’‐ TCCGCAGCACTCAGACTACG‐3’	5’‐ GGCAACAGCGTCAGAATCCA‐3’
ASIC1a	5’‐CACAGATGGCTGATGAAAAGCAG‐3’	5’‐CATGGTAACAGCATTGCAGGTGC‐3’
β‐actin	5’‐ACCACAGCTGAGAGGGAAATCG‐3’	5’‐AGAGGTCTTTACGGATGTCAACG‐3’

### 2.8 Western blot analysis

Rat liver tissues and HSC‐T6 cells were lysed with RIPA lysis buffer (Beyotime, China). After centrifugation at 12 000 g at 4°C for 30 minutes, the supernatants were collected. Proteins were mixed with Laemmli sample buffer and boiled at 100°C for 10 minutes. Extracts were separated by sodium dodecyl sulphate polyacrylamide gel electrophoresis (SDS‐PAGE, 10%, 80 V for 30 minutes and then 120 V for 60 minutes), transferred to a polyvinylidene difluoride (PVDF) membrane (Millipore, USA), blocked in TBS/Tween‐20 containing 5% nonfat dry milk at 37°C for 3 hours, washed in TBS/Tween‐20 three times, then incubated with primary antibodies against ASIC1a (1:500, Alomone, USA), a‐SMA (1:400, Bioss, China), Collagen I (1:400, Bioss, China), Caspase12 (1:1000, CST, USA), GRP78 (1:1000, CST, USA), IREI (1:1000, CST, USA), XBPI (1:1000, Elabscience, China), p‐AKT(1:1000, CST, USA) and β‐actin (1:500, Bioss, China) overnight at 4°C. The blots were washed in TBS/Tween‐20 then incubated with secondary antibodies for 1 hour. Blots were washed in TBS/Tween‐20 three times, protein bands were visualized with ECL chemiluminescent kit (ECL‐plus, Thermo Scientific).

### 2.9 Laser scanning confocal microscopy

Cells(2 × 10^5^)on coverslips were washed three times with Hank's solution and incubated with 4 μmol/L Fluo‐3‐AM and 0.02% Pluronic F‐127 (Biotium, Hayward, California, USA) for 30 minutes at 37°C. Cells were washed three times with Hank's solution at 25°C to remove the extracellular Fluo3‐AM. Nimodipine (5 μmol/L) was added to eliminate the effects of voltage‐gated Ca^2+^ channels from intracellular stores. Cells were perfused initially with D‐Hank's solution (pH6.5), PcTx1 and LY294002 with buffer containing PDGF (10 ng/ml). The fluorescence of intracellular Fluo‐3 was quantitated by confocal laser scanning fluorescence microscopy (Zeiss) with excitation at 488 nm and emission at 525 nm. Grey scale images with 0‐255 steps were collected at different time‐points before and up to 10 minutes after fluorescence microscopy and archived as TIFF image files for later analysis.

### 2.10 Statistics

Results are represented as mean ± SD. The comparison among the different treatment groups was performed with analysis of variance (ANOVA) and unpaired Student's *t* test. *P* < 0.05 is regarded as statistically significant.

## 3 RESULTS

### 3.1 ASIC1a was up‐regulated in liver tissues of patients with liver fibrosis

**Figure 1:**
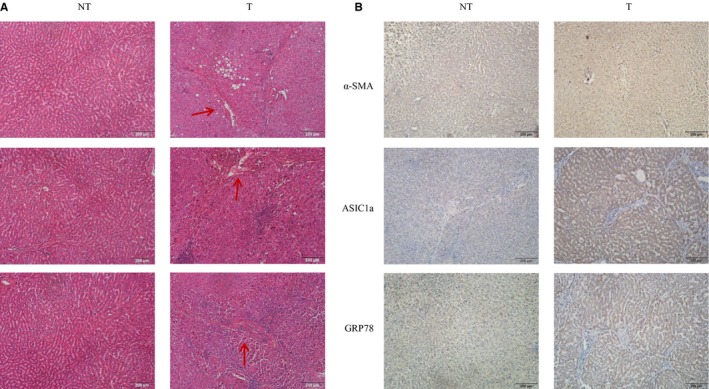
ASIC1a was up‐regulated in liver tissues of patients with liver fibrosis. A, Photomicrographs showing the liver of patients with liver fibrosis stained with hematoxylin and eosin. B, Protein levels of α‐SMA, ASIC1a and GRP78 in liver fibrosis tissues (T) and normal tissues (NT). (original magnification × 200; n(T) = 20, n(NT) = 10)

To investigate the potential function of ASIC1a in human liver fibrosis, we examined ASIC1a expression in liver tissues and normal tissues by immunohistochemical staining. We found ASIC1a was significantly up‐regulated in disease tissue compared with control (Figure 1A). In addition, we found the liver fibrosis protein marker α‐SMA and PI3K/AKT pathway proteins GRP78 and p‐AKT were significantly up‐regulated in liver tissues compared with controls **(**Figure 1B). These results suggest ASIC1a is important in the regulation of liver fibrosis by affecting ERS and the PI3K/AKT pathway.

### 3.2 ASIC1a and ERS‐related proteins were up‐regulated in rat liver tissues and PDGF‐induced HSC cells

**Figure 2:**
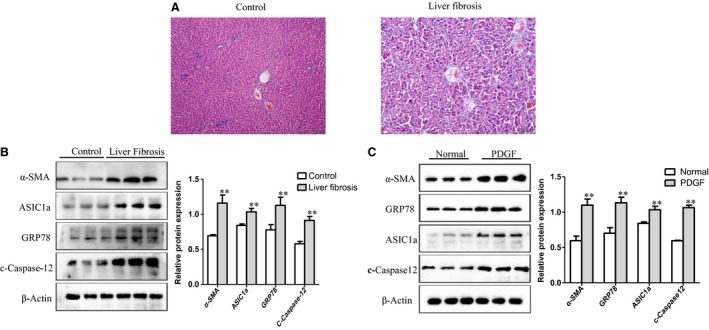
ASIC1a and endoplasmic reticulum stress‐related proteins were up‐regulated in rat liver tissues and HSC‐T6 cells. A, Photomicrographs showing the liver of the experimental rats stained with hematoxylin and eosin (original magnification × 200; n = 15). B, The expression of ASIC1a, a‐SMA, GRP78 and C‐Caspase12 in the liver tissues of experimental rats. **P < 0.01 vs Control group (n = 3). C, The expression of ASIC1a, a‐SMA, GRP78 and C‐Caspase12 in HSC‐T6 cells induced by PDGF‐BB. **P < 0.01 vs Normal group (n = 3)

We activated HSCs with PDGF‐BB(10 ng/ml, Protech, USA)and measured the effects. We found that liver tissue and liver lobule were completely intact with the normal structure and healthy arrangement of cells in the control group (Figure 2A). In comparison, in liver fibrosis tissues, we found rat hepatocytes showed steatosis, necrosis, infiltration of inflammatory cells in the more serious portal area and central vein and deposition of collagen fibres, larger fibre spacing and formation of false lobular. The results show that ASIC1a and ERS‐related protein expressions increased in liver fibrosis tissues and PDGF‐activated HSC‐T6 cells (Figure 2B, C). These data indicate that ASIC1a and ERS are involved in the progression of liver fibrosis.

### 3.3 Silencing ASIC1a attenuated the activation and proliferation of HSC‐T6 cells induced by PDGF‐BB

**Figure 3:**
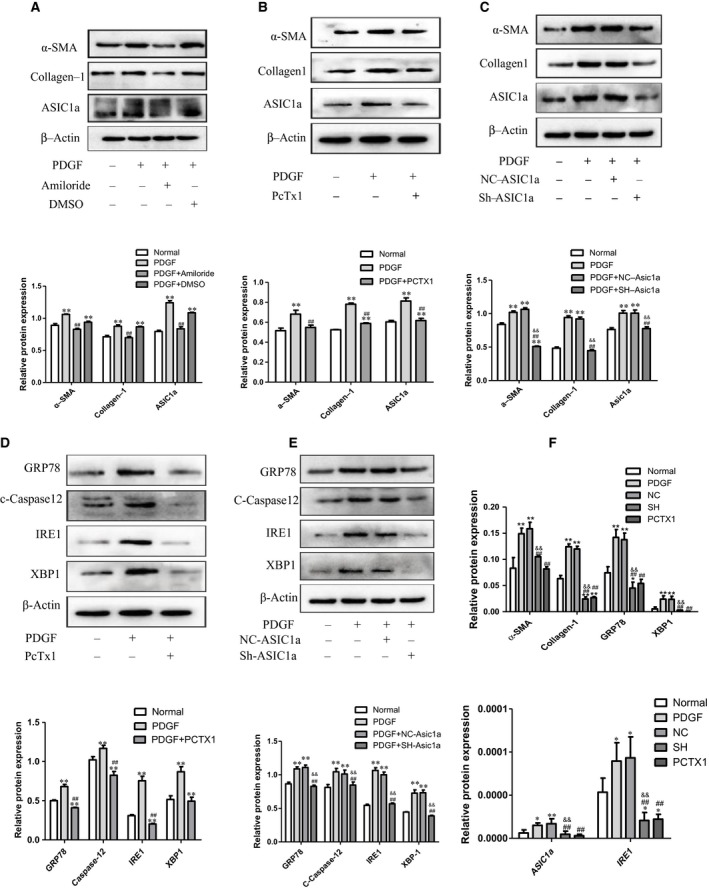
Silencing ASIC1a attenuated the activation and proliferation of HSC‐T6 cells induced by PDGF‐BB. A, B and C, Silencing ASIC1a inhibited the expression of a‐SMA and Collagen I in PDGF‐BB‐stimulated HSC‐T6 cells. **P < 0.01 vs Normal group; ^##^ *P *< 0.01 vs PDGF group; ^&&^ *P*<0.01 vs PDGF+NC‐ShRNA group. D and E, Silencing ASIC1a inhibited the expression of ERS marker proteins GRP78 and Caspase12, and IRE1‐XBP1 in PDGF‐BB‐stimulated HSC‐T6 cells. **P<0.01 vs Normal group; ^##^ *P*<0.01 vs PDGF group; ^&&^ *P*<0.01 vs PDGF+NC‐ShRNA group. F, RT‐PCR analysis showing silencing ASIC1a attenuated the activation and proliferation of HSC‐T6 (n = 3)

To investigate ASIC1a's effect on HSC‐T6 cells induced by PDGF‐BB, we silenced ASIC1a with a nonspecific inhibitor (Amiloride), a specific inhibitor (PcTx1) and ASIC1a‐ShRNA. Results show levels of α‐SMA and Collagen I significantly reduced after Amiloride, PcTx1 or ASIC1a‐ShRNA administration (Figure 3A‐C respectively). This suggests that ASIC1a participates in the activation and proliferation of HSCs.

To confirm whether ASIC1a affects ERS in HSC‐T6 cells stimulated by PDGF‐BB, we measured ERS protein markers GRP78, Caspase12 and downstream pathway marker IRE1‐XBP1 after silencing ASIC1a. We found that GRP78, Caspase12 and IRE1‐XBP1 significantly reduced after PcTx1 or ASIC1a‐ShRNA administration (Figure 3D, E respectively). Consistently, q‐PCR results align with Western blot data (Figure 3F). These results suggest that ASIC1a plays an important role in the pathogenesis of liver fibrosis through ERS related pathway.

### 3.4 PDGF activated membrane transport of ASIC1a in HSC by PI3K/AKT pathway

**Figure 4:**
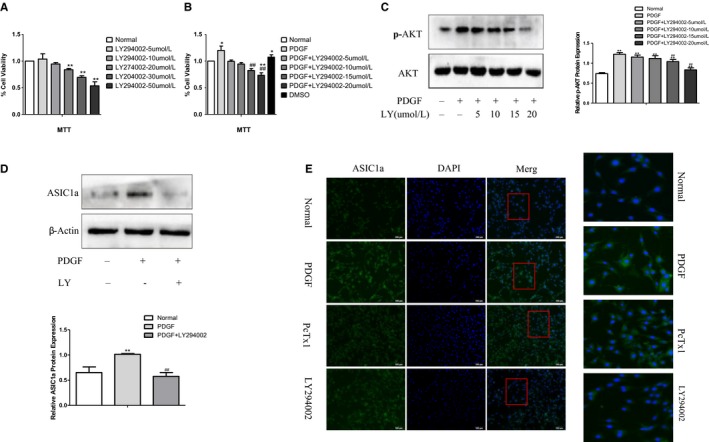
PDGF activated membrane transport of ASIC1a in HSC by PI3K/AKT pathway. A, Toxicity of PI3K/AKT inhibitor (LY294002) on liver stellate cells at different concentrations. **P<0.01 vs Normal group. B, Effect of PI3K/AKT inhibitor (LY294002) on PDGF‐stimulated liver stellate cells at different concentrations. *P<0.05,*^*^ *P*<0.01 vs Normal group; ^##^ *P*<0.01 vs PDGF group. C, Effect of PI3K/AKT inhibitor (LY294002) with different concentrations on expression of p‐AKT in PDGF‐induced liver stellate cells. **P<0.01 vs Normal group; ^##^ *P*<0.01 vs PDGF group. D, LY294002 inhibited the expression of ASIC1a in PDGF‐induced liver stellate cells. **P<0.01 vs Normal group; ^##^ *P*<0.01 vs PDGF group. E, Representative immunofluorescence analysis performed on HSCs using anti‐ASIC1a (green) antibody after PDGF treatment for 24 h. Nuclei were counterstained with DAPI. Merge contains the combined image of ASIC1a immunostaining and DAPI staining. Experiments were conducted in triplicate in three independent cell cultures (original magnification × 200) (n = 3)

To further determine the role of ASIC1a activation in liver fibrosis, we inhibited the PI3K/AKT pathway with LY294002 in PDGF‐BB induced HSC‐T6 cells. MTT assay results show that LY294002 reduced the number of HSCs (Figure 4A, B), and WB results show that LY294002 reduced the expression of p‐AKT (Figure 4C). We found LY294002 inhibited PI3K/AKT most effectively at 15 μmol/L. Therefore, this concentration was used in further experiments. We found that ASIC1a expression significantly decreased after silencing PI3K/AKT, suggesting this pathway up‐regulates the activity of the ASIC1a membrane channel (Figure 4D).

We measured the surface population of ASIC1a channels in cultured HSC‐T6 cells by immunofluorescence and found significantly more total ASIC1a, and they migrated from the nucleus and cytoplasm to the cell membrane after PDGF‐BB stimulation (Figure 4E). By contrast, blocking ASIC1a with the specific inhibitor PcTx1, or inhibiting the activation of PI3K/AKT signal pathways with LY294002 abolished this effect. This suggests the increased expression of ASIC1a and the migration is regulated via the PI3K/AKT pathway.

### 3.5 PDGF induced intracellular Ca^2+^ influx prevented by ASIC1a via PI3K/AKT pathway

**Figure 5:**
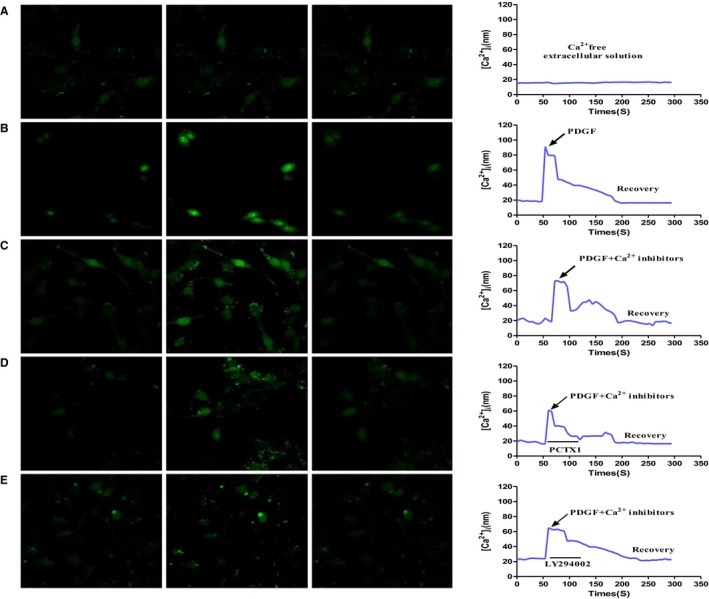
PDGF induced intracellular Ca^2 +^influx activated by ASIC1a through PI3K/AKT pathway. Cellular confocal micrographs showing changes in [Ca^2+^]_i_ concentration visualized by Fluo‐3‐AM in HSCs. A, PDGF‐induced elevation of [Ca^2+^]_i_ in Ca^2+^‐free extracellular solution. B, PDGF‐induced elevation of [Ca^2+^]_i_ in extracellular Ca^2+^ solution. C, PDGF‐induced elevation of [Ca^2+^]_i_ in HSCs treated with Ca^2+^ inhibitor. D, PDGF‐induced elevation of [Ca^2+^]_i_ in HSCs treated with PcTx1. E, PDGF‐induced elevation of [Ca^2+^]_i_ in HSCs treated with PI3K/AKT inhibitor (LY294002). (n = 3)

To further investigate PI3K/AKT signalling, we measured intracellular Ca^2+^ influx after silencing ASIC1a with LY294002 or PcTx1 in HSCs. In all experiments, voltage‐gated Ca^2+^ channels were blocked with 10 μmol/L nimodipine to remove the possibility of secondary channel activation and release of internal Ca^2+^ stores. Confocal micrograph results show PDGF significantly increased [Ca^2+^]_i_ in HSCs (Figure 5A, B). And, silencing ASIC1a with PcTx1 significantly reduced intracellular Ca^2+^ compared with PDGF group (Figure 5C). Consistently, intracellular Ca^2+^ was also reduced after PI3K/AKT inhibition with LY294002. This increased PDGF increased cytosolic Ca^2+^ concentration in HSCs by activating ASIC1a through PI3K/AKT pathway.

### 3.6 Effect of GRP78‐IRE1‐XBP1 pathway on ASIC1a expression in PDGF‐induced HSCs

**Figure 6:**
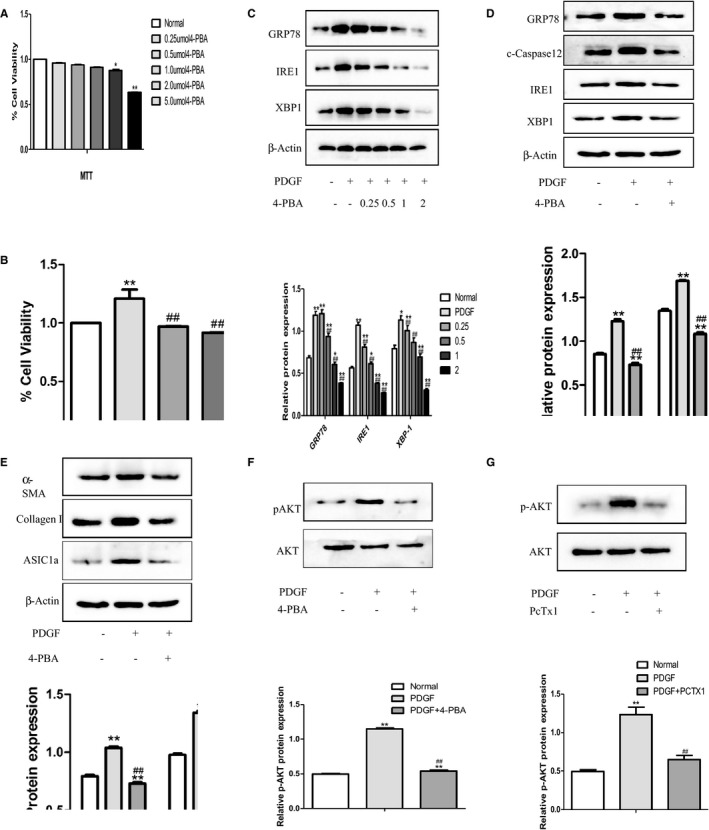
The expression of ASIC1a is related to the GRP78‐IRE1‐XBP1 pathway under endoplasmic reticulum stress. A, Toxicity of ERS inhibitor 4‐PBA on liver stellate cells at different concentrations. **P<0.01 vs Normal group. B, Effect of ERS inhibitor 4‐PBA on PDGF‐stimulated liver stellate cells at different concentrations. *P<0.05, **P<0.01 vs Normal group; ^##^ *P*<0.01 vs PDGF group. C, Effect of ERS inhibitor 4‐PBA at different concentrations on ERS‐related protein expression in PDGF‐induced liver stellate cells. **P<0.01 vs Normal group; ^##^ *P*<0.01 vs PDGF group. D, 4‐PBA inhibited the expression of ERS‐related proteins in PDGF‐induced liver stellate cells. **P<0.01 vs Normal group; ^##^ *P*<0.01 vs PDGF group. E, 4‐PBA inhibited the expression of a‐SMA, Collagen I and ASIC1a in PDGF‐induced liver stellate cells. **P<0.01 vs Normal group; ^##^ *P*<0.01 vs PDGF group. F, 4‐PBA inhibited the expression of p‐AKT in PDGF‐induced liver stellate cells. **P<0.01 vs Normal group; ^##^ *P*<0.01 vs PDGF group. G, PcTx1 inhibited the expression of p‐AKT in PDGF‐induced liver stellate cells. *^*^ *P*<0.01 vs Normal group; ^##^ *P*<0.01 vs PDGF group. (n = 3)

To further investigate the role of ASIC1a, we inhibited ERS with 4‐phenylbutanoic acid (4‐PBA), blocking the activation of Caspase‐12, GRP78 and IRE1‐XBPI pathways in PDGF‐induced HSCs. MTT assay results show toxicity of 4‐PBA on HSCs at different concentrations (Figure 6A), and effects of knockdown at differing concentrations (Figure 6B). Western blot results show the effectiveness of 4‐PBA reducing GRP78‐IRE1‐XBP1 expression (Figure 6C). We found that 4‐PBA most effectively inhibited ERS at 1 μmol/L. At this concentration, c‐Caspase12, GRP78 and IRE1‐XBP1 expression and activation were effectively silenced (Figure 6D). In addition, 4‐PBA abolished PDGF‐induced expression of ASIC1a, α‐SMA and Collagen I (Figure 6E) and reduced p‐AKT levels (Figure 6F). Finally, PcTx1 treatment also inhibited the PI3K/AKT pathway (Figure 6G). These results demonstrate a correlation between ERS and ASIC1a via PI3K/AKT signalling, where ASIC1a affects ERS‐related pathways by regulating intracellular calcium.

**Figure 7:**
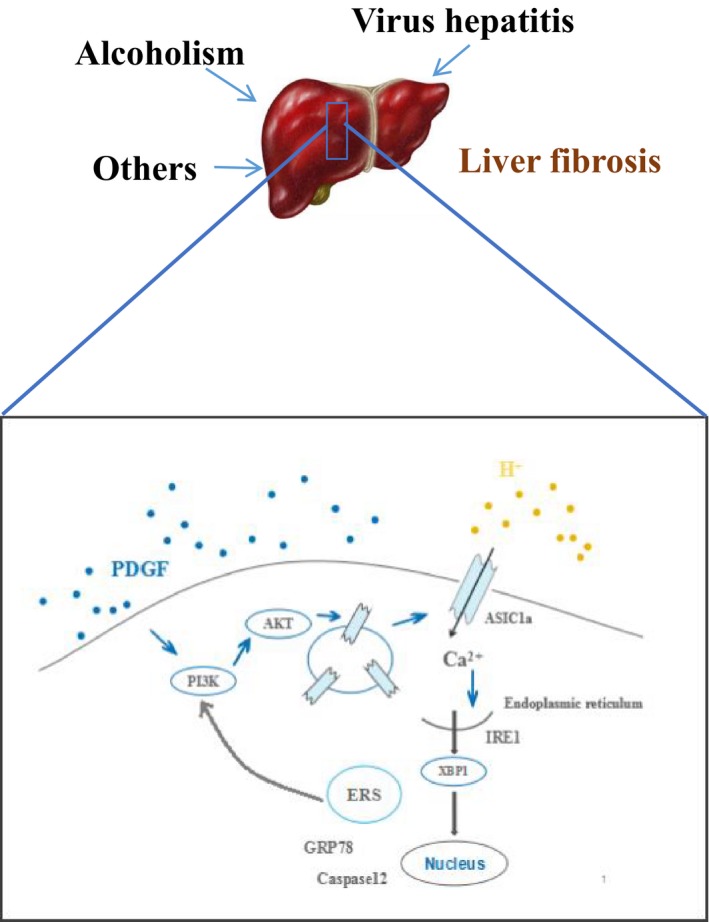
Endoplasmic reticulum stress combined with ASIC1a via PI3K/AKT pathway contributes to HSC‐T6 activation and proliferation stimulated by PDGF

## 4 DISCUSSION

Our results demonstrate ASIC1a and ERS‐related proteins were up‐regulated in CCl_4_‐induced fibrotic mouse liver tissues, PDGF‐activated HSCs and patient samples. In PDGF‐induced HSCs, ASIC1a was activated and migrated to the cell membrane, causing extracellular calcium influx that triggered ERS, which was mediated by the PI3K/AKT pathway. Calcium ions are stored in the ER and channelled through ASIC1a. Our results show ERS positively regulated ASIC1a and activated the PI3K/Akt signalling pathway, which caused ASIC channel expression and activation, Ca^2+^ influx, intracellular Ca^2+^ steady state change that induced ERS. Finally, ERS regulated PI3K/AKT pathway in a positive feedback loop.

ASIC1a is a popular research topic, mostly concentrated in the central and peripheral nervous system where expression is the highest. Few studies have reported its involvement in intracellular signalling pathways, with fewer in liver fibrosis. ASIC1a is associated with cancer and involved in the proliferation and migration of glioblastoma and breast cancer.^37^, ^38^ Jin et al. showed ASIC1a was overexpressed in liver cancer.^39^ In human liver fibrosis samples, we found high ASIC1a expression in liver tissue along with high levels of GRP78, suggesting that they participate in ERS regulation.

Our in vivo and in vitro liver fibrosis data from WB and q‐PCR demonstrated that ASIC1a regulates the expression of α‐SMA and Collagen1. Real‐time dynamic monitoring of intracellular calcium concentration by laser confocal microscopy showed [Ca^2+^]_i_ significantly increased after PDGF stimulation; this phenomenon was inhibited after pretreatment with PcTx1. We visualized the distribution of ASIC1a and membrane transport by immunocytochemistry and found increased ASIC1a that translocated from the nuclear membrane and plasma membrane to the cell membrane. Again, activation and membrane transport were inhibited by the specific inhibitor PcTx1. Youssef et al. showed ASIC1a activation is associated with the PI3K/AKT pathway.^33^, ^34^ Our data showed after treatment with the PI3K/AKT inhibitor LY294002, the ASIC1a expression and [Ca^2+^]_i_ was significantly induced in PDGF‐induced HSCs.

Our previous studies showed that ERS is involved in liver fibrosis and associated with liver inflammation. ERS‐mediated apoptosis may be a key step in activating HSCs that trigger apoptosis and promote fibrosis.^40^ We know that ERS enhances fibrosis through IRE1a‐XBP1.^36^, ^41^ Caspase‐12 is expressed in the ER, where cleavage is important for stress‐induced apoptosis.^42^ We found increased expression of ERS protein markers GRP78 and Caspase12 and IREI‐XBP1 in HSCs, indicating that IREI‐XBP1 is involved in liver fibrosis. However, we do not know how this is related to ASIC1a.

To this end, we examined the expression of ERS‐related protein after inhibiting ASIC1a. WB showed that the expressions of GRP78, c‐Caspase12 and IRE1‐XBP1 were inhibited by PcTx1 and ShRNA‐ASIC1a; q‐PCR results confirm these findings. In order to investigate whether ERS modulates ASIC1a, we used 4‐PBA as an ERS inhibitor, given its broad and effective usage. We found that GRP78, c‐Caspase12 and IRE1‐XBP1 were significantly inhibited, and α‐SMA and Collagen1 significantly decreased. Notably, ASIC1a was also reduced, which may be associated with 4‐PBA inhibition of PI3K/AKT pathway in ERS. Results showed that PcTx1 and 4‐PBA reduced the expression of p‐AKT.

In conclusion, our findings suggest ASIC1a is involved in the activation and proliferation of liver fibrosis and ERS through the PI3K/AKT pathway.

## ACKNOWLEDGEMENTS

The authors thank Z.S. WU for the help with HE and immumohistochemical staining in patients with liver fibrosis, and thank Dr. Austin Cape at ASJ Editors for careful reading and helpful suggestions.

## CONFLICT OF INTEREST

The authors declare that they have no competing interests.
